# Determination of Vascular Dementia Brain in Distinct Frequency Bands with Whole Brain Functional Connectivity Patterns

**DOI:** 10.1371/journal.pone.0054512

**Published:** 2013-01-24

**Authors:** Delong Zhang, Bo Liu, Jun Chen, Xiaoling Peng, Xian Liu, Yuanyuan Fan, Ming Liu, Ruiwang Huang

**Affiliations:** 1 Center for Studies of Psychological Application, Guangdong Key Laboratory of Mental Health and Cognitive Science, School of Psychology, South China Normal University, Guangzhou, P. R. China; 2 Department of Radiology, Guangdong Province Hospital of Traditional Chinese Medicine, Guangzhou, P. R. China; University of Cambridge, United Kingdom

## Abstract

Recent studies have shown that multivariate pattern analysis (MVPA) can be useful for distinguishing brain disorders into categories. Such analyses can substantially enrich and facilitate clinical diagnoses. Using MPVA methods, whole brain functional networks, especially those derived using different frequency windows, can be applied to detect brain states. We constructed whole brain functional networks for groups of vascular dementia (VaD) patients and controls using resting state BOLD-fMRI (rsfMRI) data from three frequency bands - slow-5 (0.01∼0.027 Hz), slow-4 (0.027∼0.073 Hz), and whole-band (0.01∼0.073 Hz). Then we used the support vector machine (SVM), a type of MVPA classifier, to determine the patterns of functional connectivity. Our results showed that the brain functional networks derived from rsfMRI data (19 VaD patients and 20 controls) in these three frequency bands appear to reflect neurobiological changes in VaD patients. Such differences could be used to differentiate the brain states of VaD patients from those of healthy individuals. We also found that the functional connectivity patterns of the human brain in the three frequency bands differed, as did their ability to differentiate brain states. Specifically, the ability of the functional connectivity pattern to differentiate VaD brains from healthy ones was more efficient in the slow-5 (0.01∼0.027 Hz) band than in the other two frequency bands. Our findings suggest that the MVPA approach could be used to detect abnormalities in the functional connectivity of VaD patients in distinct frequency bands. Identifying such abnormalities may contribute to our understanding of the pathogenesis of VaD.

## Introduction

Vascular dementia (VaD), also called multi-infarct dementia, occurs when cells in the brain are deprived of oxygen. A network of blood vessels (the vascular system) supplies the brain with oxygen. If a blockage occurs in the vascular system, or if the system is diseased, blood may be prevented from reaching the brain. As a result, cells in the brain die, leading to symptoms of dementia [1], [2]. VaD is one of the most common types of dementia, ranking second only to Alzheimer’s disease (AD) [3]. It is characterized by a sudden onset followed by a progressive decline in language, memory, and other cognitive functions. Early studies suggested that VaD is associated with a specific neuropsychological dysfunction modality that can be compared to AD [4] and other dementias [5]. The high occurrence of VaD among older adults has aroused widespread concern [3]. Although the diagnostic criteria of VaD have been continuously refined [6], more reliable, applicable diagnostic modalities continue to be urgently needed for clinical practice and research purposes.

Resting-state functional connectivity (rsFC) refers to the synchronization of neurophysiological events in spatially remote regions of the human brain in a resting state [7]. RsFC has been widely used to study various brain disorders, such as Alzheimer’s disease (AD) [8], [9], attention deficit/hyperactivity disorder (ADHD) [10], depression [11], and schizophrenia [12]. Most of these studies used a frequency band from 0.01–0.1 Hz to explore the neuronal correlates of fluctuations in fMRI signals [13]. Previous studies showed that oscillations in frequency reflect synchronized discharges by large numbers of neurons and correspond to various properties and physiological functions of the brain [14]. Buzsáki and Draguhn [14] pointed out that brain neural oscillations cover a wide range of frequencies (0.05 Hz to 500 Hz), including slow-5 (0.01∼0.27 Hz), slow-4 (0.027∼0.073 Hz), slow-3 (0.073∼0.198 Hz), slow-2 (0.198∼0.25 Hz). The architecture of functional cortical networks in the brain appears to be related to systematic neural oscillations which occur in several oscillatory bands. To distinguish the contributions of different frequency bands to regional properties of the brain state, Zuo et al. [15] and Han et al. [16] studied the distinct spatial profiles of the amplitude of spontaneous low-frequency oscillations (ALFF) in two frequency bands, the slow-5 and slow-4, and found that widespread alternations in the ALFF occurred in many brain regions and that these alterations varied widely between these two frequency bands. Several other studies [17]–[21] also investigated the effects of different frequency bands on the global properties of whole brain functional networks and brain states.

Multivariate pattern analysis (MVPA) is greatly useful for exploiting the determinative relationship between neuroimaging data patterns and categories of brain states [22]–[26]. This method has often been applied in “brain reading” research to decode specific mental states or representational information from fMRI activity patterns [27]–[29]. In such situations, MVPA tools have often been considered to be classifiers, or more generally, learning machines. MVPA enables researchers to characterize differences in brain disorders as well as to identify levels of disorder in individual brains [30], [31]. In light of these past uses, we hypothesized that rsFC patterns could be used to discriminate VaD brains from healthy brains using a MVPA method and that the abnormal functional connectivity of the rsFC patterns in VaD brains would be frequency dependent.

Our goal was to detect the frequency-specific rsFC patterns of VaD brains in order to be able to distinguish VaD brains from healthy controls. To realize this, we constructed frequency-specific brain functional networks for both the VaD patients and the controls using three different frequency bands (slow-5, slow-4, and whole-band). We then used the support vector machine (SVM), a type of MVPA classifier, on network-based rsFC measures in order to discriminate the VaD brain state from the healthy brain state. In addition, we compared the rsFC patterns corresponding to the three frequency bands to determine the differences between them with respect to their ability to discriminate the brain state of VaD patients from that of the controls.

## Materials and Methods

### Subjects

Twenty-two VaD patients (12 M/10 F) were recruited from the Department of Radiology, Guangzhou University of Traditional Chinese Medicine for this study. Table 1 lists their demographic characteristics and their primary neuropsychological information. Before the experiment, all patients had received a routine dementia investigation, including standardized clinical examinations and a conventional magnetic resonance imaging (MRI) scan. In the hours before and during the MRI scanning, the patients were not treated with any medication, in order to avoid the effect of the medications on patients. All the VaD patients were diagnosed by experienced neurologists according to the dementia criterion of DSM-IV and showed symptoms of lacunar infarcts, small white matter hyperintensities and slight brain atrophy. To avoid the impact of brain atrophy on the functional connectivity result, patients with obvious atrophy were excluded from this study. The patients were excluded as well if they met any of the following clinical characteristics: (a) classical characteristics of AD, (b) other Axis I psychiatric diagnoses, (c) serious neurologic or endocrine disorders, (d) any medical condition or treatment known to affect the brain, (e) alcohol/substance misuse related disorders, or (f) mental retardation as defined by DSM-IV criteria. Additionally, the rsfMRI data of three VaD patients (2 M/1 F) were excluded from further analysis due to excessive head motion (translation <2 mm or rotation <2°).

**Table 1:** Demographic characteristics and primary neuropsychological information of the subjects in the present study.

	VaD	Controls	*p-*value
**Gender (M/F)**	10/9	10/10	0.87
**Age (years)**	55∼81 (69.7±8.8)	57∼75 (65.4±5.0)	0.06
**Education** **(years)**	0∼13 (6.1±4.0)	0∼12 (6.5±3.7)	0.72
**MMSE**	13∼24 (18.9±3.2)	22∼30 (27.2±2.0)	1.94E−11
**MoCA**	6∼20 (12.3±4.6)	24∼30 (26.9±1.6)	1.22E−12

Data are presented as the range from min–max (mean ± SD). The *p-*value was calculated by using a two samples two-tail *t-*test. VaD, vascular dementia; MMSE, Mini-Mental State Examination; MoCA, Montreal Cognitive Assessment (Beijing version).

To make a between-group comparison, we also recruited from the local community a control group that was comprised of twenty healthy participants who were age- and gender-matched to the VaD group. All subjects (both the VaD patients and the controls) were right-handed and finished a standardized clinical evaluation protocol, including the mini-mental state exam (MMSE) and the Montreal Cognitive Assessment (MoCA), the results of which are also listed in Table 1. We found significant differences in the clinical evaluation scores of the MMSE and MoCA, but no significant differences in age, gender, and years of education between the two subject groups. This study was approved by the Institutional Review Board of Guangzhou University of Traditional Chinese Medicine. Written informed consent was obtained from each participant or a family member (legal guardian) prior to the experiment.

### Data Acquisition

All participants were scanned on a 1.5T Siemens Avanto MR scanner with a 12-channel phased-array head coil. During the data acquisition, each participant was asked to lie quietly in the MR scanner with their eyes closed, but to stay awake and try not to think about anything. The rsfMRI data were acquired using a gradient-echo echo-planar imaging (GE-EPI) sequence. The sequence parameters were as follows: repetition time (TR) = 2000 ms, echo time (TE) = 39 ms, ﬂip angle = 90°, FOV = 240 mm×240 mm, data matrix = 64×64, slice thickness = 4 mm, interslice gap = 1 mm, 30 slices along the AC-PC line covering the whole brain, and 180 volumes. We also acquired high resolution 3D brain structural images using a T1-weighted MP-RAGE sequence (TR = 1160 ms, TE = 4.21 ms, TI = 900 ms, flip angle = 15°, FOV = 256 mm×256 mm, matrix = 256 mm×256 mm, slice thickness = 1 mm, and 192 sagittal slices).

### Data Preprocessing

Data preprocessing was performed using SPM8 (http://www.fil.ion.ucl.ac.uk/spm/) and DPARSF (http://www.restfmri.net/forum/DPARSF). For each participant, the first 10 volumes of rsfMRI data were discarded to reduce the effects of signal equilibrium and the participant’s adaptation to the scanning noise. The remaining rsfMRI data were corrected for the intra-volume acquisition time delay between slices and then normalized into the Montreal Neurological Institute (MNI) space by applying the EPI template at a 3×3×3 mm^3^ resolution. We removed linear trends in the process of preprocessing. Also, we regressed out the covariates of head motion (About head motion information, see Table S1) and signals from the whole brain and CSF as well as those from the white matter. No spatial smoothing was performed on the rsfMRI datasets during preprocessing because spatial averaging might blur out fine-grained spatial patterns [32]. Similar to previous studies [15], [16], we obtained the waveform for each voxel to reduce low-frequency drift and high-frequency physiological noise in three different frequency bands, 0.01∼0.27 Hz (slow-5), 0.027∼0.073 Hz (slow-4), and 0.01∼0.073 Hz (whole-band).

### Construction of Brain Functional Networks

The brain was parcellated into 90 cortical regions of interest (ROIs) according to the Automated Anatomical Labeling (AAL) template [33]. Table 2 lists the name and the abbreviations of the brain regions used in this study.

**Table 2:** List of brain regions extracted from the Automated Anatomical Labeling (AAL) template (Tzourio-Mazoyer et al., 2002) and their abbreviations as used in this study.

Index	Regions	Abbreviation
(1,2)	Precental gyrus	PreCG
(3,4)	Superior frontal gyrus, dorsolateral	SFGdor
(5,6)	Superior frontal gyrus, orbital part	ORBsup
(7,8)	Middle frontal gyrus	MFG
(9,10)	Middle frontal gyrus, orbital part	ORBmid
(11,12)	Inferior frontal gyrus, opercular part	IFGoperc
(13,14)	Inferior frontal gyrus, triangular part	IFGtriang
(15,16)	Inferior frontal gyrus, orbital part	ORBinf
(17,18)	Rolandic operculum	ROL
(19,20)	Supplementary motor area	SMA
(21,22)	Olfactory cortex	OLF
(23,24)	Superior frontal gyrus, medial	SFGmed
(25,26)	Superior frontal gyrus, medial orbital	ORBsupmed
(27,28)	Gyrus rectus	REC
(29,30)	Insula	INS
(31,32)	Anterior cingulate and paracingulate gyri	ACG
(33,34)	Median cingulate and paracingulate gyri	MCG
(35,36)	Posterior cingulate gyrus	PCG
(37,38)	Hippocampus	HIP
(39,40)	Parahippocampal gyrus	PHG
(41,42)	Amygdala	AMYG
(43,44)	Calcarine fissure and surrounding cortex	CAL
(45,46)	Cuneus	CUN
(47,48)	Lingual gyrus	LING
(49,50)	Superior occipital gyrus	SOG
(51,52)	Middle occipital gyrus	MOG
(53,54)	Inferior occipital gyrus	IOG
(55,56)	Fusiform gyrus	FFG
(57,58)	Postcentral gyrus	PoCG
(59,60)	Superior parietal gyrus	SPG
(61,62)	Inferior parietal, but supramarginal and angular gyri	IPL
(63,64)	Supramarginal gyrus	SMG
(65,66)	Angular gyrus	ANG
(67,68)	Precuneus	PCUN
(69,70)	Paracentral lobule	PCL
(71,72)	Caudate nucleus	CAU
(73,74)	Lenticular nucleus putamen	PUT
(75,76)	Lenticular nucleus, pallidum	PAL
(77,78)	Thalamus	THA
(79,80)	Heschl gyrus	HES
(81,82)	Superior temporal gyrus	STG
(83,84)	Temporal pole: superior temporal gyrus	TPOsup
(85,86)	Middle temporal gyrus	MTG
(87,88)	Temporal pole: middle temporal gyrus	TPOmid
(89,90)	Inferior temporal gyrus	ITG

The same 45 brain regions were extracted from the right and left hemispheres to provide 90 regional time series in total for each subject.

For each of the three frequency bands (slow-5, slow-4, and whole-band), we first obtained a time series for each ROI by averaging the time courses of all the voxels in each of the participant’s ROIs and then calculated the Pearson’s correlation coefficient between any pair of ROIs. In this way, a 90×90 functional connectivity matrix was determined for each subject. This matrix included both negative and positive correlation values [34]. Within a given frequency band for each participant, a brain functional connectivity matrix was constructed using each cortical ROI as a node and the Pearson’s correlation coefficient between any pair of nodes as the weight of the edge.

**Figure 1:**
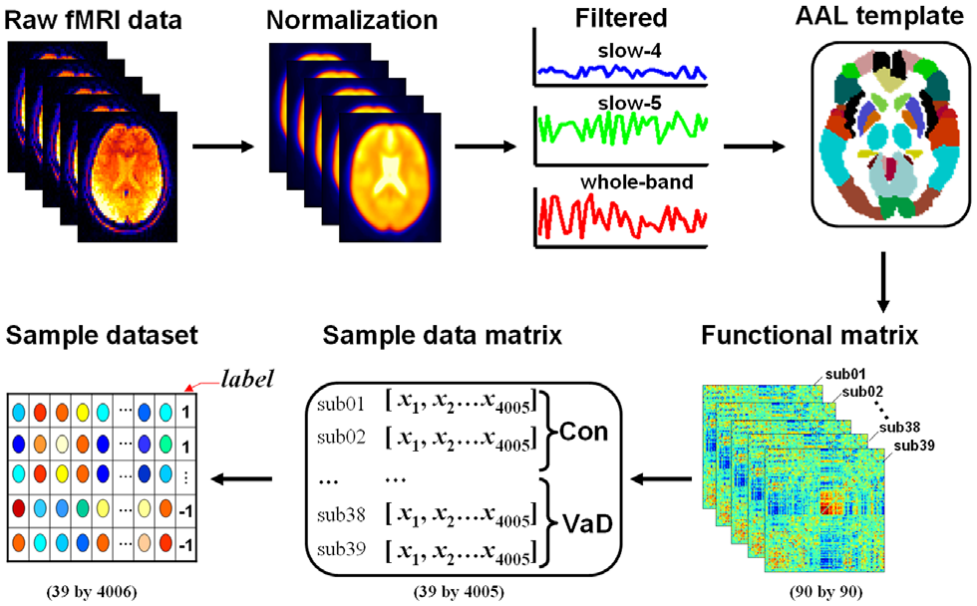
The preprocessing procedure used in the construction of the sample dataset based on resting state fMRI (rsfMRI) data in three frequency bands. The rsfMRI data were normalized to the MNI standard space using the EPI template and filtered using 0.01∼0.027 Hz (slow-5), 0.027∼0.073 Hz (slow-4), and 0.01∼0.073 Hz (whole-band) frequencies. Brain regions were defined according to the AAL atlas, and the time series was extracted for each region. The whole brain functional network was constructed for each frequency band by taking each cortical region as a node and the inter-regional Pearson’s correlation coefficient as the edge for each subject. All the independent elements of the individual functional connectivity matrix (90×90) were arranged into a 1-by-4005 row vector. We assembled all the row vectors for all 39 subjects into a 39-by-4005 matrix and normalized the data using their z-scores. The normalized matrix and the subject labels (patients were labeled by 1s and healthy subjects as -1s) constituted the sample dataset for further SVM analysis. In total, we obtained three sample datasets corresponding to the three different frequency bands.

Fig. 1 shows the procedure for constructing the sample dataset. Within a given frequency band (e.g., the slow-5), we extracted *N* (*N*-1)/2 = 4005 independent elements from each individual brain functional connectivity matrix and arranged them into a row vector (1 x 4005). *N = *90 was the number of ROIs used to construct the brain functional networks. By assembling the row vectors from all 39 subjects, we obtained a 39×4005 matrix, which was used as the sample dataset in this study. In this way, we built three sample datasets to correspond to the three frequency bands - slow-5, slow-4, and whole-band. In each sample dataset, we added a label of 1 or -1 to every row of the sample dataset to indicate whether it corresponded to a VaD patient or to a healthy subject, respectively. The sample dataset, that is, the 39×4005 matrix, was normalized using a *r*-to-*z* transform (the Fisher z transform) [35]

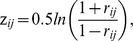
where *r*_ij_ represents one measurement of inter-regional functional connectivity, i.e., a single element of the sample dataset. The normalized sample dataset was used for the remaining calculations except where stated otherwise.

### Pattern Classification

Previous studies have indicated that SVM methods are reliable and less sensitive to noise than other methods when used to separate brain states into groups [36]. In this study, we used the LIBSVM classifier (http://www.csie.ntu.edu.tw/wcjlin/libsvm) to separate the brains of VaD patients from those of the healthy controls.

**Figure 2:**
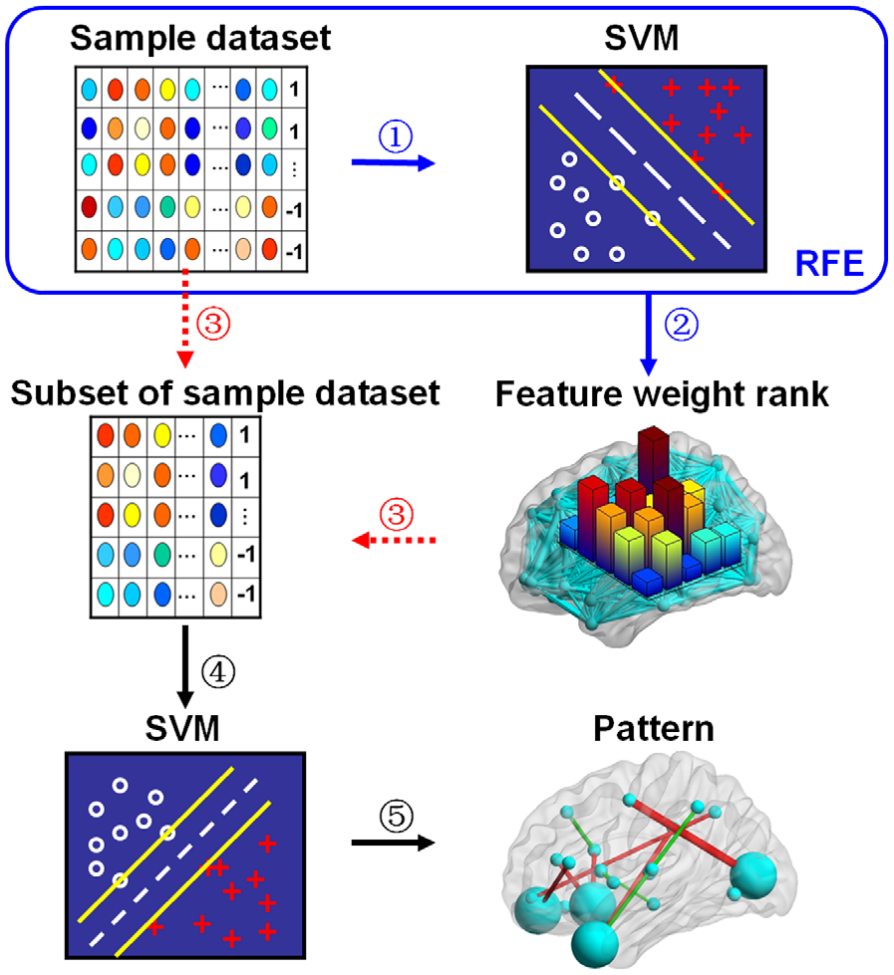
The flow chart of the rsFC pattern classification obtained by applying MVPA to the sample dataset in the slow-5 frequency band. The sample dataset was a 39-by-4005 matrix in this study and was comprised of 4005 features, that is, inter-regional functional connections. Each row and each column of the sample dataset represented a subject and a feature, respectively. Recursive feature elimination (RFE) was applied to the sample dataset for feature selection and to calculate the rank of the feature weight (a row vector). The subsets of the sample dataset were produced according to the feature weighting rank. The classifier was used to separate the VaD brains from those of the controls in each subset of the sample dataset by using a leave one-out-subject-cross-validation (LOOCV). The accuracy rate was used to compare the discriminative information in each subset of the sample dataset.

Fig. 2 shows the three steps of the rsFC pattern classification in the slow-5 frequency band. Step-1: Feature selection. We took each inter-regional functional connection as a feature. For the sample dataset, the 39×4005 matrix, we thus have 4005 features. This step involved selecting the features which had appropriate information for performing the rsFC classification [29], [35]. We first applied a recursive feature elimination (RFE) method [37] to weight the features and arrange them in rank order according to their weight, that is to their contribution to the pattern classification. Then we extracted the subsets of the sample dataset that related to the slow-5, also arranging them according to the feature ranking and obtained 4005 subsets of the sample dataset. The number of subsets of the sample dataset is equal to the number of features. In detail, the first subset was a 39×1 matrix corresponding to the first feature; the second subset was a 39×2 matrix which corresponded to ranking of the first two features in declining order, and so on. The 4005th subset was a 39×4005 matrix that contained all of features, which is the sample dataset itself, only arranged in declining order by weight. Note that all of the subsets had the same number of rows, i.e. the number of total subjects, but the number of columns was equal to the number of features.

Step-2: Pattern classification. We evaluated the performance of the SVM classifier using each of the subsets to validate the classifier performance. The default parameters of the LIBSVM classifier were adopted in the calculations. For each subset of the data sample, the leave-one-subject-out-cross-validation (LOOCV) method [35], [38] was applied to evaluate the performance of the classifier. Given *K* subjects (in this study, *K* = 39), we split the subset of the data sample into *K*-folds, each subject corresponding to a fold. We used *K*-1 folds to train the classifier and the remaining one to test the classifier. The LOOCV procedure iterated until each fold was left out one time. In total, the LOOCV process was iterated *K = *39 times and each of iterations produced a corresponding determination accuracy. In the end, we estimated the determination accuracy rate by averaging all of the accuracies achieved from every fold of the determination.

Step-3: Information comparison of the pattern classification. In order to select the subset of the sample dataset with the maximum discriminative information, we compared the determination accuracy rates that corresponded to each subset of the sample dataset. The subset that obtained the highest accuracy rate was identified and used for further analysis.

We used the same calculation procedure for the other two frequency bands, the slow-4 and whole-band. In the end, we obtained three selected subsets from the sample dataset based on the classification convergence, each subset corresponding to one frequency band.

## Results

**Figure 3:**
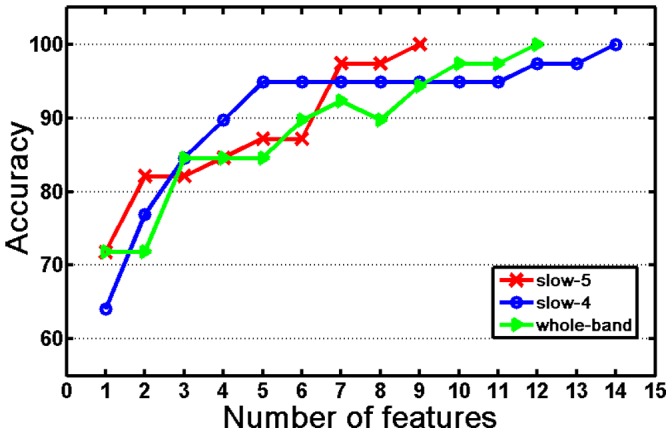
Graph of the convergence of the accuracy rate with the number of features in the pattern classification subsets. Triangles in red, green, and blue show how the accuracy rates changed with the number of features in the slow-5, slow-4, and whole-band frequency bands, respectively.

Fig. 3 shows the tendency for the determination accuracy rates to change with the number of selected features before converging. We found that the determination accuracy rates showed a consistent change with an increase in feature number. The accuracy rates increased initially and then reached convergence, that is, leveled off, for any given frequency band. Determination accuracy rates in all three frequency bands reached convergence with 100% accuracy. The speeds of convergence, that is, the number of iterations needed to obtain convergence, in the three frequency bands differed, with the slow-5 band showing the fastest convergence speed.

In order to examine whether the high determination accuracy rates resulted from overfitting the classifier, we re-validated the SVM classifier with the selected subset data under the condition that everything was kept in correspondence with the above analysis, but the subject labels (1 and −1) were randomized. When we did this, we obtained accuracy rates of 56.4%, 38.46%, and 58.9% corresponding to the slow-5, slow-4, and whole-band, respectively. This indicates that the determination accuracy rates when the labels were randomized were approximately random, indicating that the 100% accuracy in the classification result shown in Fig. 3 indeed reflects actual group differences in the rsFC patterns between the VaD patients and the controls.

**Figure 4:**
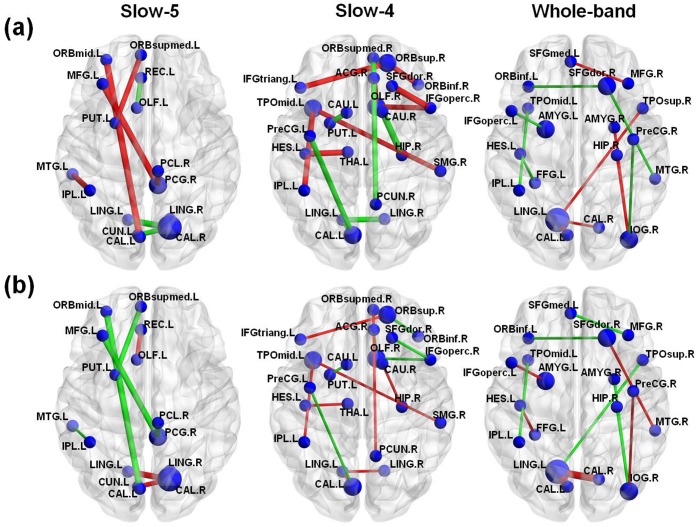
The selected features, that is, the inter-regional functional connections in the resting state functional connectivity pattern classification for the VaD group and the control group in the three frequency bands. Upper panel: The features selected in the VaD patients. The node size is proportional to the frequency occurrence of the brain region in the selected features and the line thickness to the mean value of the feature. The red (green) lines represent features that were increased (decreased) in the VaD group compared with the controls. Lower panel: Same as the upper panel but for the control group. For both panels: slow-5∶0.01∼0.27 Hz (low frequency); slow-4∶0.027∼0.073 Hz (high frequency); whole-band: 0.01∼0.073 Hz.

Fig. 4 shows the selected features for the VaD group and for the control group in the rsFC pattern classification at the point where the accuracy rates reached convergence for the slow-5, slow-4 and whole-band frequency bands. The slow-5 band reached convergence with the fewest number of features, nine, whereas the slow-4 reached convergence with the greatest number of features, fourteen. Table 3 shows that fifteen, twenty-two, and nineteen brain regions were involved in the features that were selected for pattern classification in the slow-5, slow-4, and whole-band frequency bands, respectively. These brain regions are widely distributed in the frontal cortex, temporal lobe, parietal lobe, visual cortex, and subcortical regions. Three brain regions, the CAL.L, IPL.L, and LING.L, appeared among the selected features for all three frequency bands.

**Table 3:** Brain regions involved in the features selected from the functional connectivity pattern for differentiating the brain states of the VD patients from those of the controls in the three frequency bands.

	Regions		
Index	slow-5	slow-4	whole-band
1	**CAL.L**	**CAL.L**	**CAL.L**
2	**IPL.L**	**IPL.L**	**IPL.L**
3	**LING.L**	**LING.L**	**LING.L**
4	**LING.R**	**LING.R**	IOG.R
5	**PUT.L**	**PUT.L**	MFG.R
6	MFG.L	**HES.L**	**HES.L**
7	CUN.L	**HIP.R**	**HIP.R**
8	MTG.L	**SFGdor.R**	**SFGdor.R**
9	OLF.L	**TPOmid.L**	**TPOmid.L**
10	**CAL.R**	ACG.R	**CAL.R**
11	ORBmid.L	CAU.L	AMYG.L
12	ORBsupmed.L	CAU.R	AMYG.R
13	PCG.R	IFGoperc.R	FFG.L
14	PCL.R	IFGtriang.L	IFGoperc.L
15	REC.L	OLF.R	MTG.R
16	–	ORBinf.R	ORBinf.L
17	–	ORBsup.R	PreCG.R
18	–	ORBsupmed.R	SFGmed.L
19	–	PCUN.R	TPOsup.R
20	–	PreCG.L	–
21	–	SMG.R	–
22	–	THA.L	–

The brain regions in bold type appeared in all three frequency band subsets of the sample dataset. The abbreviations of the brain regions and their coordinates in the MNI standard space can be found in Table S1.

Table 4 shows statistical comparisons between the VaD patient group and the control group for each feature selected in the rsFC pattern classifications. In the pattern classification the selected features are listed in the order of their feature weight from high to low. Both the feature weight in the pattern classification and the number of selected features differed between the three frequency bands. We found nine, fourteen, and twelve features for the slow-5, slow-4, and whole-band frequency bands, respectively. For the slow-5 frequency band, we found that five features decreased and four increased in the VaD group compared with the control group. The features that decreased were inter-regional connections between the IPL.L and the MTG.L, between the ORBmid.L and the CUN.L, between the OLF.L and the REC.L, between the CAL.L and the CAL.R, and between the CAL.R and the LING.R, whereas the features that increased were the inter-regional functional connections between the MFG.L and the PCG.R, between the ORBsupmed.L and the PUT.L, between the PCG.R and the PCL.R, and between the LING.L and the CAL.R. Four of these features were located in the left hemisphere, three features linked both hemispheres, but only two features were located in the right hemisphere. For the slow-4 frequency band, the SVM calculations selected fourteen features that were able to discriminate each individual VaD brain from the healthy ones. Five of these features were in the left hemisphere, three features linked both hemispheres, and six features were located in the right hemisphere. Specially, among the fourteen features, we detected four significantly increased features (inter-regional connections between the SFGdor.R and IFGoperc.R, between the IPL.L and TPOmid.L, between the SMG.R and TPOmid.L, and between the THA.L and HES.L) and three decreased features (inter-regional connections between the HIP.R and CAU.R, between the LING.L and LING.R, and between the CAU.L and PUT.L). For the whole–band frequency, we detected six features that decreased (inter-regional connections between the LING.L and the CAL.R, between the MTG.R and the SFGdor.R, between the FFG.L and the HES.L, between the AMYG.L and the IFGoperc.L, between the IOG.R and the PreCG.R, and between the CAL.L and the LING.L) and six features that increased (between the ORBinf.L and the SFGdor.R, between the HIP.R and the IOG.R, between the SFGmed.L and the MFG.R, between the AMYG.R and the IOG.R, between the LING.L and the TPOsup.R, and between the IPL.L and the TPOmid.L) in the VaD group compared with the control group. Four features were located in the left hemisphere, another four features linked the two hemispheres, and the remaining four features were located in the right hemisphere. In the three frequency bands, we detected several decreased inter-regional functional connections that were located in vision-related regions (in the slow-5: between the CAL.R and the LING.R, between the CAL.R and the LING.L, and between the CAL.L and the CAL.R; in the slow-4: between the LING.L and the LING.R and between the CAL.L and the LING.L; in the whole-band: between the CAL.R and the LING.L and between the CAL.L and the LING.L).

**Table 4:** Statistical comparisons between the selected features of the VaD patients and the controls in the three frequency bands.

	Features (Inter-regional functional connections)		Group mean value of each feature (std)		
Frequency bands	Regions	Regions	VaD	Controls	*t*-value
**slow-5**	IPL.L	MTG.L	0.41 (0.41)	−0.17 (0.26)	−3.14^**^
	MFG.L	PCG.R	0.47 (0.25)	−0.44 (0.24)	3.17^**^
	CUN.L	ORBmid.L	0.49 (0.29)	−0.47 (0.14)	−2.73^**^
	OLF.L	REC.L	−0.36 (0.35)	0.34 (0.33)	−3.37^**^
	ORBsupmed.L	PUT.L	0.39 (0.24)	−0.37(0.29)	2.34^*^
	PCG.R	PCL.R	0.48 (0.39)	−0.46 (0.44)	2.30^*^
	CAL.L	CAL.R	−0.37 (0.32)	0.35 (0.09)	−2.52^*^
	LING.L	CAL.R	−0.47 (0.36)	0.45 (0.33)	2.36^*^
	CAL.R	LING.R	−0.36 (0.34)	0.35 (0.24)	−3.30^**^
**slow**−**4**	CAL.L	PreCG.L	−0.40 (0.20)	−0.15 (0.16)	−0.99
	IFGoperc.R	SFGdor.R	0.47 (0.24)	−0.01 (0.23)	2.43^*^
	IFGtriang.L	ORBsup.R	0.41 (0.26)	0.04 (0.18)	−1.51
	ORBinf.R	ORBsup.R	0.40 (0.25)	−0.03 (0.24)	−1.17
	IFGoperc.R	OLF.R	0.39 (0.19)	−0.10 (0.11)	1.62
	ACG.R	OLF.R	0.44 (0.22)	−0.17 (0.21)	0.04
	ORBsupmed.R	PCUN.R	−0.39 (0.24)	0.06 (0.17)	−1.21
	CAU.R	HIP.R	−0.49 (0.26)	0.08 (0.22)	2.17^*^
	CAL.L	LING.L	−0.37 (0.21)	0.02 (0.23)	0.61
	LING.L	LING.R	−0.40 (0.24)	0.04 (0.21)	−2.56^*^
	IPL.L	TPOmid.L	0.42 (0.22)	0.06 (0.20)	−2.97^**^
	TPOmid.L	SMG.R	0.37 (0.22)	0.01 (0.16)	2.56^*^
	CAU.L	PUT.L	−0.40 (0.24)	−0.02 (0.17)	−2.65^*^
	HES.L	THA.L	0.43 (0.32)	0.08 (0.22)	2.58^*^
**Whole**-**band**	CAL.R	LING.L	0.17 (0.23)	0.54 (0.18)	4.50^**^
	ORBinf.L	SFGdor.R	−0.01 (0.18)	−0.18 (0.21)	−3.00^**^
	HIP.R	IOG.R	0.05 (0.26)	−0.15 (0.23)	−3.75^**^
	MTG.R	SFGdor.R	−0.05 (0.17)	0.13 (0.15)	2.70^*^
	MFG.R	SFGmed.L	0.16 (0.14)	−0.08 (0.20)	−3.30^*^
	FFG.L	HES.L	−0.04 (0.18)	0.09 (0.19)	2.52^*^
	AMYG.R	IOG.R	0.15 (0.25)	−0.08 (0.19)	−3.71^**^
	AMYG.L	IFGoperc.L	−0.09 (0.21)	0.08 (0.20)	3.44^**^
	LING.L	TPOsup.R	0.05 (0.18)	−0.15 (0.19)	−3.10^**^
	IOG.R	PreCG.R	−0.01 (0.14)	0.19 (0.19)	2.91^**^
	IPL.L	TPOmid.L	−0.03 (0.19)	−0.19 (0.19)	−3.14^**^
	CAL.L	LING.L	0.35 (0.22)	0.65 (0.29)	4.30^**^

Group mean value for each selected feature was calculated by averaging the feature values, the inter-regional functional connections, across all subjects in the VaD group or in the control group. Positive or negative feature values represent inter-regional functional correlations or anticorrelations, respectively. Statistical comparisons of the feature values between the VaD patients and the controls were estimated based on a two samples *t*-test. A positive (negative) *t*-value stands for a feature that was significantly increased (decreased) in the VaD group compared with the control group. The features are listed in order of decreasing weighted rank in the pattern discrimination. The symbol “**” represents significant difference determined by *p*<0.01 and “*” determined by *p*<0.05.

## Discussion

This study investigated the possibility of using pattern classification to distinguish individual VaD brains from healthy ones, based on whole brain functional networks in three different frequency bands. Using a high-dimensional pattern classification concept, we utilized an SVM classifier to analyze whole brain functional networks and to select features for differentiating the VaD brain from that of healthy controls in terms of their rsFC patterns. The determination accuracy was estimated using the LOOCV process to ensure the stability of the discrimination. The results indicated that the abnormal functional connectivity in the rsFC patterns depended on the specific frequency band, which reflects the frequency-specific spatiotemporal information distribution of the BOLD signal. Further analysis indicated that the slow-5 frequency band was more efficient than the slow-4 and than the whole-band for discriminating the VaD brain from the controls when using an SVM classifier.

### Dynamic Changes in the Pattern Information

A fundamental question in the MVPA approach is how to select the features that have the maximum discriminative information to use in constituting the patterns [27], [29], [35]. In most of the previous studies that used MVPA, the feature selection was quite static in that the features were selected once and then not reconsidered [22], [23]. In this study, we constructed the rsFC patterns by using the features, that is, the inter-regional functional connections, based on feature weighting, and then used an SVM classifier to determinate the brain category based on these rsFC patterns. The determination accuracy rates were used to assess the magnitude of the pattern information [28]. Fig. 3 shows the dynamic changes in the determination accuracy rates with the number of features required to obtain convergence. Specifically, the accuracy rate increased with an increase in the number of selected features. For example, in frequency band slow-5 (Fig. 3), the accuracy was about 82% when three features were selected, was about 97% with seven selected features, and was 100% with nine selected features. The results indicate that, within any given frequency band, the more features contained in the connectivity patterns, the more neurobiological information involved in the corresponding patterns in VaD brains. This provides further evidence that functional connectivity can reveal VaD neurobiological information.

### Frequency Bands Influence Pattern Discriminative Information

In this study, we proposed that we could obtain a frequency-specific description of brain functional networks by decomposing the band-pass filtered time series into smaller frequency intervals. Fig. 3 indicates that the fastest convergence speed occurred in the slow-5 frequency band. Thus, the connectivity pattern of the slow-5 frequency exhibited a greater discriminative power than the other two frequency bands (slow-4 and whole-band). In addition, the accuracy of the classification improved using the slow-5 band compared with the conventional whole-band approach. Although the convergence accuracy rates were same at 100%, the number of features that were selected from the rsFC patterns in order to accurately discriminate those individuals with VaD brains differed between the three frequency bands.

We also found that the numbers and the locations of the selected features in the pattern classification differed between the three frequency bands. We recognized nine, fourteen, and twelve features in the rsFC pattern discrimination and these features occurred in fifteen, twenty-two, and nineteen brain regions (Table 3) for the slow-5, slow-4, and whole-band frequency bands, respectively. The distributions of the selected features in the slow-5 (low frequency) and in the slow-4 (high frequency) were quite different (Fig. 4). For the slow-5, four features were located in the left hemisphere, two features were in the right hemisphere, and three features linked both hemispheres. For the slow-4, we found five features in the left hemisphere, six features in the right hemisphere, and three features linking both hemispheres.

The values of the selected features in the VaD group and the control group differed between the three frequency bands (Table 4). The mean value of each feature was calculated across all subjects for each subject group. For the slow-5 band, we noticed that the mean value (absolute value) of each feature was nearly the same for the VaD group (0.36∼0.49) and for the control (0.17∼0.47), but the direction of each feature for the VaD group was opposite to that of the control (Table 4). However, for the slow-4 band, the mean value of each feature in the VaD group was much larger than that of the control group. For the whole-band, the mean value of each feature in the VaD group was much larger than that of the control group except for two features, LING.L-CAL.L and LING.L-CAL.R. We found that most of selected features in the rsFC patterns were different for the three frequency bands.

Differences in lateralization (left vs. right hemispheres) of the feature distribution were observed between the slow-5 and slow-4 frequency bands. We found that the brain regions related to the features selected for the slow-5 band were primarily located in the left frontal cortex and bilateral visual cortex and were obviously asymmetric (Fig. 4). However, the regions of the selected features for the slow-4 band were located in the right frontal cortex and left temporal lobe as well as in the bilateral visual cortex. This may indicate that the left frontal cortical regions involve primarily low frequency (slow-5) wavelengths but the right frontal cortex involve high frequency (slow-4) wavelengths of spontaneous activity in the resting state of VaD brains.

This study suggests that the discriminative information in an rsFC pattern depends on specific frequency bands. Several previous studies have examined the rsfMRI dataset by decomposing the BOLD signal into different frequency bands [15], [17]–[21] and have demonstrated that the power to analyze brain functional properties varies between different frequency bands. The reliability of frequency-specific spatiotemporal structures has also been tested [15]. Consistent with these previous studies, our study indicated that brain spontaneous BOLD fluctuations exhibit frequency-specific spatiotemporal patterns in whole-brain functional networks derived from BOLD-based rsfMRI datasets.

### Selected Features and Abnormal Connections in the VaD Brain

The present study showed that rsFC can be used to discriminate VaD brains from healthy ones by using the MVPA approach. The selected features (Table 4 and Fig. 4) indicated the presence of abnormal functional connections in the VaD patients compared with the controls in the three frequency bands. These abnormal inter-regional functional connections may reflect the pathogenesis of VaD brain function.

We observed decreased functional connectivity in the VaD group in the vision related regions (CAL, IPL, and LING) in all three frequency bands (Table 2). This may indicate that spontaneous activation in the visual cortex is independent of the frequency band, even though the visual cortex had substantial levels of spontaneous activation, especially in the primary visual and related regions. This result was consistent with a previous study, in which Salvador et al. [18] used inter-regional mutual information to measure brain functional connectivity in different frequency domains and showed that the occipital cortex had substantial levels of mutual information at low and high frequencies, especially in the primary visual and related regions, such as the calcarine and lingual cortices.

In this study, we also found significantly increased functional connectivity in terms of the intensity in the VaD brains in the three frequency bands (Fig. 4 and Table 4). This may reflect compensation by the brain for the functional disconnection in the visual cortex of the VaD patients. We detected increased inter-regional connections in the frontal cortex for the slow-4 band (Fig. 4). This finding was consistent with previous studies of cognitive decline with age in older adults [39], which suggested that age-related reduction in occipital activity was coupled with age-related increased frontal activity. According to the opinion of Grady and collaborators [40], [41], the increased frontal region activity in older adults may be an attempt to compensate for sensory processing deficits in the occipitotemporal regions. For the slow-5 band, we detected increased functional connectivity to the regions of the default-mode network (DMN) (Table 4), which is also consistent with previous studies [42], [43]. Attenuated deactivations of the DMN have been shown in several populations, including Alzheimer’s [44] and amnesia [45]. For patients with VaD, the reduced cerebral blood ﬂow (CBF) may influence the BOLD fMRI signal [46] causing abnormal DMN connectivity, such as the functional connectivity between the left middle frontal gyrus (MFG.L) and the right posterior cingulate gyrus (PCG.R) which were observed in the present study.

This result may provide a novel insight into the decline in multiple cognitive functions in VaD patients. The cause of the VaD symptoms may not only lie in the abnormal functional connectivity but also in compensatory adaptations in the brain systems [39]. The brain functions of VaD patients may be damaged extensively by the blood vessel infarction [47]. The changes could include executive and visuospatial dysfunction, fluctuations in attention, visual hallucinations, language problems, delusions and confusion [48] and memory related problems [39], [49], [50]. We found that the brain regions that have been selected for accurate detection of individuals with VaD include parts of the prefrontal cortex [50], [51], medial temporal lobe [52], [53], parietal [54], [55], and occipital regions [40], [56], as well as various subcortical regions [57], [58]. Previous neuroimaging studies [52], [58], [59] have suggested that brain function decline, especially memory-related functional decline, is accompanied by widely distributed focal neuronal activity changes in these brain regions, and the symptoms of VaD relate to the failure to integrate down-up information from the sensory cortex and top-down expectations from the high level cortex. Our method was blind to prior knowledge about the brain regions associated with VaD, yet the selected regions coincided well with those reported in the literature. Previous studies have indicated that most cognitive functions require the active participation of multiple cortical areas rather than a simple focal brain region [59]–[61]. The present study provided evidence that the rsFC in a VaD brain is widely disrupted compared to the rsFC in a normal, healthy brain and that the compensatory adaptations reflected in the rsFC patterns vary between frequency bands.

### Frequency-specific Whole Brain Functional Networks

We found that each of selected features corresponded to significantly change inter-regional connections in the VaD brain in the slow-5 and whole bands (Table 3). But in the slow-4 band, we obtained fourteen features for the rsFC pattern classification, but only seven appeared to represent abnormal connections in the VaD brains (Table 4). We noticed that not only the selected features but also the significantly changed inter-regional connections in the VaD brains were different in the three frequency bands. Fig. 4 and Table 4 show that most of the abnormal connections in the VaD brains were located in the left hemisphere for the slow-5 band, in the frontal cortex and the visual cortex for the slow-4 band, and in both the hemispheres for the whole-band frequency. This suggests that alterations in the whole brain functional network in VaD brains are also frequency specific.

### Limitations of the Present Study

Several limiting factors need to be addressed. The somewhat small sample size in this study may have been an issue. Additionally, a possible overlap between early AD and VaD in our patients cannot be completely excluded. Since VaD and AD are both common in old age, they can co-occur and may have among our patients. Therefore, unsurprisingly, our results are fairly consistent with previous findings involving AD [17], [22]. Another issue is that all of the VaD patients had lacunar infarcts, small white matter hyperintensities, significant grey matter reduction, and potential grey matter volume atrophy. The existence of grey matter volume atrophy in the VaD brains may affect the normalization and parcellation which could have a potential impact on the brain functional connectivity [62] and on our results. How to reduce the potential confounds stemming from brain anatomical differences or registration errors is a fundamental issue in functional studies of patients with brain atrophy [63], [64].

In the present study, we have tried several measures to reduce the influence of the brain atrophy on the ability to use patterns to discriminate VaD brains from healthy ones. First of all, we excluded patients that showed obvious atrophy. Then, in the data preprocessing, we regressed out whole brain, white matter, and CSF signals as covariates. Further, during the realignment process in order to normalize the rsfMRI data into the MNI space, we compared two different co-registration methods: a two step co-registration, in which we first registered the rsfMRI data to the T1-weighted brain structural images and then registered them to the MNI-152 template, and a one step co-registration, in which we registered the rsfMRI data to the EPI template. After comparing the co-registrations, we chose the one step process and applied the EPI template to normalize the functional data into the MNI space. In fact, several previous studies have applied the registration with the EPI template in the brain network studies of stroke [65], major depressive disorder (MDD) [66], Alzheimer’s disease (AD) [67], and schizophrenia [68]. In most of these studies, the brain atrophy was observed. Similar to these previous studies, we found that the EPI template was also appropriate for performing the functional images registration in the VaD brains. Although the classifier we trained using the method reported in this paper performed well by efficiently discriminating VaD patients from the controls, the influence of the co-registration method on the result must be considered in future studies.

Another potential limitation is that the final results might have been influenced by the way we defined the nodes and edges of the functional networks. We constructed the whole brain functional networks using the AAL template to define the nodes and inter-regional Pearson’s correlation coefficients as the weight of the edges. However, several different brain templates, such as the Automatic Nonlinear Imaging Matching and Anatomical Labeling (ANIMAL) [69] and the Harvard-Oxford atlas (HOA) [70], can be used to define nodes, and several different ways, including partial correlations, wavelet correlations, and mutual information, can be used to define edges [71]. Using different definitions of nodes and edges could influence the selected features in the rsFC pattern classification. In this study, we constituted ROI-based large-scale functional networks rather than voxel-based functional networks. This could potentially reduce the accuracy of the determination due to the above mentioned co-registration errors. Last, but not least, during data processing we performed ROI-based averaging without performing any spatial smoothing in order to preserve the spatial information in the BOLD signal. Although the question remains open as to whether spatial smoothing needs to be performed or not in MVPA, this study did not compare the influence of spatial smoothing on the determination accuracy.

In summary, we proposed a data-driven SVM method to distinguish VaD patients from normal controls. The results suggest that whole brain functional networks may be able to provide neurobiological information about VaD brain states. Thus, the SVM classifier was very useful in distinguishing the VaD brain state from the healthy brain state based on altered neurobiological information. To our knowledge, this study was the first to compare the contributions of different frequency bands to brain functional networks and their component features in VaD patients. It also showed that the neurobiological information contained in the brain functional networks is frequency-dependent. Our findings, obtained using the MVPA approach, revealed the necessity of detecting VaD brain oscillations in different frequency bands apart from the whole band. The slow-5 (0.01∼0.027 Hz) frequency window showed greater discriminatory power in separating the VaD patients from the controls. How other frequency bands affect the organization of functional networks and the rsFC pattern classification needs further study. Although exploring the nature of abnormal functional connectivity in VaD brains will need more direct evidence, our findings suggest that functional connectivity can offer frequency-specific neurobiological information about VaD patients and that this study was helpful for increasing the understanding of the neural mechanisms in VaD.

## Supporting Information

Table S1 **Head motion parameters in vascular dementia (VaD) patients and healthy controls.**
(DOC)
