# Correction: Determination of Vascular Dementia Brain in Distinct Frequency Bands with Whole Brain Functional Connectivity Patterns

**DOI:** 10.1371/annotation/f1fda637-4c81-4e7e-948e-12ffd833c352

**Published:** 2013-06-07

**Authors:** Delong Zhang, Bo Liu, Jun Chen, Xiaoling Peng, Xian Liu, Yuanyuan Fan, Ming Liu, Ruiwang Huang

There was an error in the Materials and Methods section of the PDF in the fourth sentence of the paragraph labeled "Data Acquisition". The correct sentence is, "The sequence parameters were as follows: repetition time (TR) =2000 ms, echo time (TE) =39 ms, flip angle =90°, FOV=240 mm×240 mm, data matrix =64×64, slice thickness = 4 mm, interslice gap= 1 mm, 30 slices along the AC-PC line covering the whole brain, and 180 volumes." The XML version is correct.

In addition, the email address for the third corresponding author is incorrect. The correct email address is: ruiwang.huang@gmail.com. 

