# Supplementary material for: Determination of Vascular Dementia Brain in Distinct Frequency Bands with Whole Brain Functional Connectivity Patterns
Source: PLoS One. 2013 Jan 24;8(1):e54512. doi: 10.1371/journal.pone.0054512 (PMC3554744; doi:10.1371/journal.pone.0054512)
Supplement: Table S1 — Head motion parameters in vascular dementia (VaD) patients and healthy controls. (DOC) [file pone.0054512.s001.doc]

**Table S1.** Head motion parameters in vascular dementia (VaD) patients and healthy controls.

| **Control** | Translation (mm) | | | | Rotation (degree) | | | |
| --- | --- | --- | --- | --- | --- | --- | --- | --- |
| Subject | x | y | z | norm | pitch | roll | yaw | norm |
| 1 | 0.188 | 0.061 | 0.411 | 0.457 | 0.149 | 0.342 | 0.469 | 0.599 |
| 2 | 0.201 | 0.164 | 0.392 | 0.470 | 0.714 | 0.317 | 0.186 | 0.804 |
| 3 | 0.148 | 0.071 | 0.249 | 0.298 | 0.368 | 0.178 | 0.079 | 0.416 |
| 4 | 0.118 | 0.212 | 0.328 | 0.408 | 0.156 | 0.280 | 0.137 | 0.349 |
| 5 | 0.399 | 0.212 | 0.452 | 0.640 | 0.880 | 0.491 | 0.620 | 1.184 |
| 6 | 0.058 | 0.109 | 0.336 | 0.358 | 0.130 | 0.054 | 0.038 | 0.146 |
| 7 | 0.114 | 0.109 | 0.225 | 0.275 | 0.141 | 0.066 | 0.115 | 0.194 |
| 8 | 0.175 | 0.119 | 0.246 | 0.325 | 0.332 | 0.061 | 0.123 | 0.359 |
| 9 | 0.274 | 0.172 | 0.334 | 0.465 | 0.275 | 0.346 | 0.100 | 0.454 |
| 10 | 0.083 | 0.120 | 0.254 | 0.293 | 0.357 | 0.145 | 0.068 | 0.392 |
| 11 | 0.103 | 0.149 | 0.158 | 0.240 | 0.122 | 0.049 | 0.024 | 0.134 |
| 12 | 0.256 | 0.186 | 0.262 | 0.411 | 0.210 | 0.288 | 0.158 | 0.390 |
| 13 | 0.163 | 0.120 | 0.216 | 0.296 | 0.803 | 0.375 | 0.185 | 0.906 |
| 14 | 0.245 | 0.236 | 0.297 | 0.452 | 0.705 | 0.096 | 0.258 | 0.757 |
| 15 | 0.052 | 0.113 | 0.518 | 0.533 | 0.245 | 0.079 | 0.122 | 0.285 |
| 16 | 0.325 | 0.238 | 0.689 | 0.799 | 0.583 | 0.364 | 0.154 | 0.705 |
| 17 | 0.127 | 0.096 | 0.266 | 0.311 | 0.189 | 0.164 | 0.147 | 0.291 |
| 18 | 0.101 | 0.099 | 0.252 | 0.289 | 0.117 | 0.230 | 0.074 | 0.269 |
| 19 | 0.096 | 0.175 | 0.172 | 0.264 | 0.669 | 0.165 | 0.067 | 0.693 |
| 20 | 0.063 | 0.238 | 0.536 | 0.590 | 0.248 | 0.299 | 0.191 | 0.434 |
| **VaD** | Translation (mm) | | | | Rotation (degree) | | | |
| Subject | x | y | z | norm | pitch | roll | yaw | norm |
| 1 | 0.406 | 1.122 | 0.220 | 1.210 | 1.835 | 0.401 | 0.397 | 1.920 |
| 2 | 0.198 | 0.265 | 0.417 | 0.533 | 1.470 | 0.447 | 0.288 | 1.564 |
| 3 | 0.152 | 0.347 | 0.504 | 0.631 | 0.313 | 0.191 | 0.199 | 0.418 |
| 4 | 0.130 | 0.132 | 0.174 | 0.255 | 0.169 | 0.046 | 0.140 | 0.224 |
| 5 | 0.312 | 0.127 | 0.468 | 0.577 | 0.466 | 0.215 | 0.303 | 0.597 |
| 6 | 0.927 | 0.222 | 0.282 | 0.994 | 1.643 | 0.446 | 0.761 | 1.865 |
| 7 | 0.124 | 0.188 | 0.749 | 0.782 | 0.630 | 0.160 | 0.064 | 0.653 |
| 8 | 0.103 | 0.164 | 0.176 | 0.262 | 0.184 | 0.098 | 0.227 | 0.309 |
| 9 | 0.545 | 0.243 | 0.320 | 0.678 | 0.156 | 0.234 | 0.475 | 0.553 |
| 10 | 0.140 | 0.197 | 0.736 | 0.775 | 0.859 | 0.232 | 0.401 | 0.976 |
| 11 | 0.673 | 0.225 | 0.378 | 0.804 | 0.357 | 0.536 | 0.923 | 1.126 |
| 12 | 0.159 | 0.250 | 0.899 | 0.947 | 0.626 | 0.995 | 0.814 | 1.430 |
| 13 | 0.890 | 0.170 | 0.450 | 1.012 | 1.093 | 0.1360 | 0.293 | 1.140 |
| 14 | 0.151 | 0.111 | 0.129 | 0.228 | 0.465 | 0.248 | 0.160 | 0.551 |
| 15 | 0.046 | 0.285 | 0.327 | 0.436 | 2.670 | 0.673 | 0.491 | 2.797 |
| 16 | 0.309 | 0.131 | 0.191 | 0.387 | 0.184 | 0.289 | 0.168 | 0.382 |
| 17 | 0.124 | 0.103 | 0.114 | 0.198 | 0.476 | 0.257 | 0.224 | 0.586 |
| 18 | 0.012 | 0.162 | 0.565 | 0.588 | 0.209 | 0.132 | 0.134 | 0.282 |
| 19 | 0.083 | 0.372 | 0.350 | 0.518 | 1.032 | 0.144 | 0.251 | 1.072 |
